# The Effect of Task-Oriented Activities Training on Upper-Limb Function, Daily Activities, and Quality of Life in Chronic Stroke Patients: A Randomized Controlled Trial

**DOI:** 10.3390/ijerph192114125

**Published:** 2022-10-29

**Authors:** Abdulrahman M. Alsubiheen, Wonho Choi, Wonjong Yu, Haneul Lee

**Affiliations:** 1 Department of Rehabilitation Sciences, College of Applied Medical Sciences, King Saud University, Riyadh 11495, Saudi Arabia; 2 Department of Physical Therapy, College of Health Science, Gachon University, Incheon 21936, Korea; 3 Department of Physical Therapy, Eulji University, Seongnam 13135, Korea

**Keywords:** activities of daily living, chronic stroke, task-oriented ADL training, upper-limb function, quality of life

## Abstract

This randomized controlled study aimed to investigate the effects of 8-week task-oriented activities of daily living (T-ADL) training on upper limb functions, activities of daily living (ADL), and quality of life (QoL) in chronic stroke patients. The 33 patients were randomly assigned to the T-ADL training or conventional occupational therapy (OT) group. The respective interventions were provided for 45-min a day, five times a week for eight weeks. To compare the upper-limb function before and after the intervention, the manual function test (MFT), box and block test (BBT), and grasp power test were performed; to compare the level of ADL performance, the modified-Barthel index (MBI) was measured. To evaluate QoL, stroke-specific QoL was measured. There was a significant group-by-time interaction in the affected side MFT score and both sides of BBT scores, but no significant interaction was found in the unaffected side MFT score, ADL, and QoL. Both groups showed a significant main effect of time in their ADL and QoL after the intervention (*p* < 0.001). The results of this study indicate that the eight-week T-ADL training has a positive effect on upper limb functions and gross manual dexterity, and both T-ADL training and conventional OT are effective in improving ADL and QoL in chronic stroke patients.

## 1. Introduction

Recently, with the prolongation of mean human life expectancy, problems related to population aging and the incidence of adult vascular diseases have increased. Moreover, the incidence of stroke as a type of vascular disease in younger adults has increased owing to various factors [1,2,3]. The World Health Organization (WHO) has reported an annual global incidence of 15 million stroke patients. Stroke is also known as a neurological defect that causes damage to the cerebrovascular system with symptoms of reduced blood supply to the brain, such as thrombosis, embolism, and hemorrhage [3,4,5].

Notably, only 10% of post-stroke patients show complete recovery, 30% die, and 60% suffer from chronic dysfunction [6]. Among those suffer from chronic dysfunction, 60–80% of patients experience functional dyskinesia of the upper extremity as a result of stroke [7,8]. These patients perform daily activities mainly using the unaffected upper limb, and as they avoid using the affected limb [9,10], the end result is the complete disuse of the affected limb [9]. Since most small-scale activities, such as eating, washing, getting dressed, and writing, involve the hands and upper limbs, the inability to use an upper limb could cause loss of upper limb function and, consequently, lead to a reduction in the motion related to daily living to prevent independent activities of daily living (ADL) [11]. This could increase feelings of depression, anxiety, sleep deprivation, and helplessness, transform the overall lifestyle toward dependency, cause a reduction in self-respect and self-efficacy, and induce physiological pain [12], thereby affecting the quality of life (QoL) [13].

A task-oriented approach is an activity-centered approach that involves the repeated training of a task focusing on functional performance toward the effective completion of the task, whereby the exercise training effects are increased through the environment, task analysis, feedback, and repeated training [14]. Functional tasks, which help restore the reflex loops into a network of neural CNS patterns, help to organize motor behavior, whereas occupational performance allows interactions in various environmental systems built around human characteristics and their environments. Behavioral change is induced in a patient after a change in the human or environmental system. Based on this theory, as the patient attempts to accomplish a functional goal, the effectiveness of the approach lies in providing the patient with a functional task rather than a training of the pattern of normal motion, thereby providing a chance for the patient to actively attempt to solve problems. In addition, the patient performs various task-oriented activities through effective therapy that includes various functional activities [15]. Compared to the practice of tasks designed in a clinical setting, the functional performance of tasks related to actual daily living is more useful, which occurs in the task-oriented approach as a clinically applicable intervention for patients [15]. Thus, the treatment aims to improve motor functions as patients actively use the upper limb on the affected side [10].

Recent research has investigated the effects of depression, rehabilitation motivation, and ADL on the QoL of stroke patients [16]. Other researchers conducted a study based on ADL in task-oriented training to investigate the effects of task-oriented training of the upper extremities on upper-limb function and ADL in stroke patients and the effects of task-oriented activity monitoring training on the dexterity of the affected limb, ADL, and brainwave patterns [17,18]. However, among the various studies applying a task-oriented approach, only a few studies have focused on the independent performance of ADL by the patient in task-oriented training across various and extensive categories of daily living and its effect on functional and QoL improvements. Thus, this study aimed to determine the effects of task-oriented ADL (T-ADL) training on upper-extremity functional recovery, ADL, and QoL in chronic stroke patients with related hemiplegia.

## 2. Methods

### 2.1. Study Design and Ethical Approval

This randomized controlled trial was conducted in accordance with the Consolidated Standards of Reporting Trial (CONSORT) recommendations and retrospectively registered on cris.org (KCT0007830). The study was conducted in accordance with the guidelines of the Declaration of Helsinki and approved by the Gachon University Institutional Review Board (1044396-202112-HR-241-01). All participants signed an informed consent form before the start of the study.

### 2.2. Participants

Thirty-eight chronic stroke patients were assessed for eligibility in the current study. Among these, four did not meet the inclusion criteria, and one did not participate; thus, 33 patients were included in the study. The inclusion criteria of the study participants were as follows: Hemiplegia due to stroke six months after onset. The following types of patients were excluded: patients with cognitive impairment or dementia with a Korean version of the Mini-Mental State Examination score of 23 or lower who were unable to understand instructions or inability to understand oral instruction due to language disorders or other reasons, and patients with severe contracture due to orthopedic disease of the shoulder, elbow, and wrist joints. 

### 2.3. Procedure

Thirty-three patients were randomly assigned to the T-ADL training group and the control group, with 15 patients assigned to each group using simple randomization methods that were independently conducted. Concealed allocation was performed using a computer-generated randomized table of numbers before data collection. 

All participants in both groups underwent a manual function test (MFT), box and block test (BBT), handgrip strength test, Korean version of the modified Barthel index (K-MBI), and stroke-specific QoL (SS-QoL) evaluation for baseline assessment. Both groups underwent their respective interventions for 45 min daily, five times weekly, for eight weeks (a total of 40 times). Then, all assessments were done after the 8 weeks after the intervention.

### 2.4. Outcome Measures

The primary outcomes in the study were changes in upper limb functions, measured using the MFT and BBT. The MFT was developed to measure the motor function of the affected upper limb in stroke [19]. The MFT consisted of 3 questions on arm motions (*n* = 4), grasp (*n* = 2), and hand activities (*n* = 2). It is easily applicable as an objective test for the recovery of upper-limb functions and ADL at the level of practice. Notably, this simple and easy test allows for the testing of upper limb function in stroke patients within only 10 min [20]. The BBT is used to evaluate upper-limb gross manual dexterity for frequent ADL [21]. The test requires the patient to move 150 blocks of 1 inch from one box to another, and the score is based on the number of blocks moved by each hand in one minute [21]. The test–retest reliability is 0.99 for the left-hand and was 0.94 for the right-hand. The inter-rater reliability for the right-hand was 1.00 and for the left-hand was 0.99, indicating a very high correlation [22].

The secondary outcomes included ADL and QoL. The level of ADL in stroke patients was measured in this study using the K-MBI, a revised and complemented form of the Barthel index to evaluate the independence of daily living in patients with a chronic disease [23]. Higher scores indicate a higher level of independence in ADL on a scale of 0–100. Ten questions were asked about personal hygiene, bathing, meals, toileting, climbing stairs, getting dressed, defecation and urinary control, gait (or wheelchair propulsion), and moving to a chair or bed [24]. QoL in stroke patients was measured using the SS-QoL tool developed by Williams et al. [25]. Since the tool was developed for stroke patients to include even categories that may be overlooked in a general assessment of QoL, it consisted of 12 categories and 49 questions regarding energy, family roles, language, mobility, mood, personality, self-care, social roles, thinking, upper extremity function, vision, and work/productivity. Each question was answered on a 5-point scale. The total score ranges between 49 and 245, with higher scores indicating higher QoL. In a study applying the tool to stroke patients, the reliability was reported to be high (α = 0.98) [26]. 

### 2.5. Interventions

Based on the ADL and Instrumental-ADL (IADL) items defined in the domain and process of the Occupational Therapy Practice Framework (OTPF3) published in 2002 by the American Occupational Therapy Association, the task-oriented activities and tasks reported in previous studies were examined, revised, and complemented, and the T-ADL training program in this study was constructed (Table 1). The patient listened to the explanation from the therapist and was self-prepared to perform each item of ADL in the given order. Notably, in an unavoidable circumstance that prevented performance in a given order, the order may have changed under the agreement between the patient and therapist. Before the subsequent session, the therapist described the results of the evaluation of the patient. Subsequently, through the intervention of the therapist, suggestions were made regarding the safest and most efficient movements for the patient to perform on their own. At the end of each training session, the patients underwent evaluation and intervention. All ADLs were performed by the patient themselves, and the therapist intervened only upon detecting a hazardous situation. 

The conventional OT provided in this study was based on the OTPF3 [27]. In conventional OT, the focus is on functional movements with the exclusion of swallowing. The therapy included passive joint exercise to reduce the spasticity of the affected upper extremity and increase joint range of motion. The treatment mainly involved upper-limb activities using tools such as skates, sanding boards, grahamizers, and pegboards. Depending on the recovery level of the participants, two to three tasks were selected and performed during the intervention. 

All interventions were applied by an experienced OT for 45-min for five times a week for 8 weeks. 

### 2.6. Statistical Analysis

All data were calculated as mean and SDs. Shapiro–Wilk tests were used to test the normal distribution of the data. Chi-squared and independent t-test were used to compare the general participant characteristics using the homogeneity test between the groups. 2 × 2 (group × time) repeated measures analysis of variance (RM ANOVA) was conducted to assess the group-by-time interaction effect of interest. When a significant interaction was found, a Bonferroni corrected post hoc test was conducted to compare the outcome variables between pre- and post-intervention in each group. The level of statistical significance was set at alpha = 0.05.

## 3. Results

Of the thirty-three participants, 3 participants dropped out of the study (one in the T-ADL training and two in the conventional OT group). Therefore, 30 patients completed the study. There were no significant differences in general characteristics and baseline function between patients who dropped out and those who completed the study (*p* > 0.05). The general characteristics and baseline function were comparable between the groups. The baseline characteristics of the participants are presented in Table 2.

**Figure 1:**
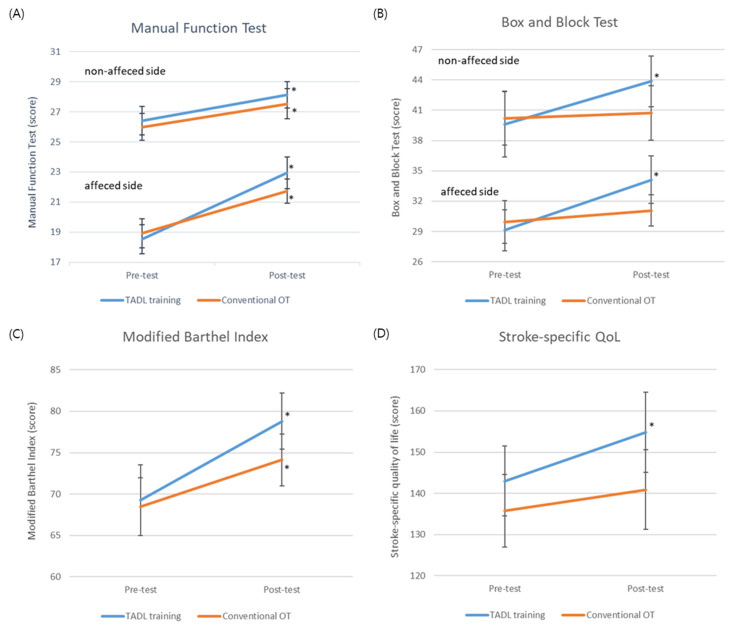
Primary and secondary outcomes at pre and post intervention (**A**) Manual function test, (**B**) Box and block test (**C**) Modified Barthel index, (**D**) Stroke-specific quality of life. Abbreviations: TADL, task-oriented activities of daily living; OT, occupational therapy; QoL, quality of life * significantly changes from pre-test to post-test.

Table 3 describes the MFT and BBT before and after the interventions in both groups to show the upper limb function. There was a significant interaction in the mean MFT score in affected side between time and group (F = 4.200, *p* = 0.050). The MFT score in affected side significantly increased after the intervention in both groups (*p* < 0.001). However, no significant interaction was found in the MFT score in non-affected side (F = 0.085, *p* = 0.773) (Figure 1A). There was a significant interaction in both side BBT score between the group and time (F = 4.411, *p* = 0.045 vs. F = 4.388, *p* = 0.045, respectively). The both side of BBT scores improved significantly from baseline to post-intervention in the T-ADL training group (*p* < 0.001), but conventional OT showed no significant improvement (*p* > 0.05) (Figure 1B). 

No significant interaction was found between the K-BMI and K-SS-QoL scores (Table 4). The K-MBI score was significantly increased after the intervention in the both groups (F = 27.27, *p* < 0.001), but the mean difference between the pre- and post- intervention in the T-ADL training group was 9.53 which can be considered as the minimal clinically important difference (MCID) for a stroke patient while the changes after the intervention in the conventional OT group was not considered as MCID (Figure 1C) [28]. Similarly, the K-SS-QoL score was significantly increased after the intervention in the both groups (F = 10.10, *p* = 0.004) (Figure 1D). 

## 4. Discussion

Stroke is a neurological disease that often causes chronic disability and permanently alters the patient’s life. Therapeutic application in stroke patients should include activities with set goals and treatments for the improvement of upper-limb functions to comprehensively address the various psychological and social problems that patients encounter in the performance environment as well as the factors related to QoL [29]. Therefore, the present study evaluated the effects of T-ADL training on upper extremity function and QoL compared to conventional OT in patients with chronic stroke.

This study found that the T-ADL training was effective in improving upper extremity function and gross manual dexterity in chronic stroke patients, with the T-ADL training group showing significantly greater increasing in MFT and BBT score compared to conventional OT group. In a recent systematic review [30], a program on ADL through task-oriented activities led to improvements in BBT and MFT scores after the intervention. Using the 10-s test, dexterity among the upper-limb functions was shown to have improved on the affected side, which was consistent with the results in this study despite the variation in the method of evaluation. In the present study, the program used in the training involved more realistic, meaningful, and active tasks on the premise. Therefore, the patients performed a task based on ADL, and the results were presumed to reflect this by the improvements in upper-limb functions with general functional improvements. Regarding the recovery of upper limb functions, recovery should be achieved in both the proximal and distal regions, while most patients rely heavily on the use of the unaffected upper limb for activities requiring the upper extremity [9,31]. 

Unlike the lower extremity, where functional movement is possible only through the use of both limbs upon an ADL, the activities related to the upper extremity mostly allow performance even through the use of a single limb, so that the use of the affected limb is frequently avoided despite residual functions to delay recovery [32]. In a situation where the compensatory use of the unaffected limb prevents the functional recovery of the affected limb, the T-ADL training in this study is presumed to have improved the upper-limb gross manual dexterity on both the affected and unaffected sides in hemiplegic patients. As the upper-limb gross manual dexterity among the physical functions have a significant impact on the independent ADL performance and prognosis of rehabilitation, T-ADL training in this study will be essential for the functional recovery of the upper extremity in stroke patients, whereas conventional OT did not affect the both upper-limb gross manual dexterity after the intervention while motor function was improved in affected upper limb. This might be because conventional OT was benefit for the gross motions but not enough for the fine motion since the manual dexterity requires both gross and fine hand motions and coordination [33]. 

A significant improvement in ADL was found in both groups, but only the T-ADL training group improved more than the MCID of the MBI. ADL can be divided into basic ADL, which covers everyday activities for consistent personal daily living and self-management, and IADL, which reflects the techniques to support home and community-based daily living, such as financial management, meal preparation, dishwashing, shopping, and transportation, and to mediate interactions with the complex environment [34]. The T-ADL training in this study comprised the items of the basic ADL (an area of occupation) as well as the IADL so that the patient could achieve optimum independence in daily living and maximum functional recovery, which was in contrast to conventional OT that focused solely on upper-limb functional movements. This was presumed to be the reason for the stronger effects of T-ADL training on the improvement of ADL performance. Additionally, consistent with our findings, previous research reported improved ADL performance through a task-oriented program for the upper extremity that included activities such as pushing the switch [15]. Stroke patients were shown a video of daily activities, such as opening and closing the refrigerator door and other activities in the kitchen, and were then guided to imitate the activities, which was reported to have improved their ADL performance [35]. The therapeutic interventions based on various training programs were shown to be more effective than simple occupational therapy focused on upper extremity training regarding an improvement in ADL performance, which coincided with the results of this study. As post-stroke patients suffer from persistent after-effects, rehabilitation for these patients should focus on the recovery of physical function as well as QoL with a consistent interest in the patients’ long-term prognosis. Our result showed that the QoL of stroke patients significantly improved in both groups. 

The findings of this study suggest that the T-ADL training program led the patients in the respective groups to show greater significant improvements in upper extremity function and gross manual dexterity. Furthermore, the affected and unaffected sides showed significantly improved upper-limb functions, which implies the importance of training focused on various ADL in not only the life at the hospital but also the discharge and return home of stroke patients. 

The strength of this study was that T-ADL was composed of task-oriented activities and it was performed by themselves based one the instructions of the therapist, which emphasizing active intervention. Despite this strength, the current study had several limitations. The sample size was small, as only those with fair cognitive abilities were selected. Thus, it is difficult to generalize the results of the T-ADL training program to all stroke patients because of the lack of varied subjects. In the future, studies should recruit a larger number of subjects and assess the performance at each stage of the program to allow generalization of T-ADL training. Further studies should be conducted on a larger number of stroke patients regarding more varied ADL as part of the rehabilitation program.

## 5. Conclusions

The results of this study indicate that the eight-week T-ADL training have a positive effect on upper limb functions and gross manual dexterity. Thus, the T-ADL training might be a feasible intervention for improving upper limb functions for both affected and non-affected side in chronic stroke patients. However, a further study with a larger sample size needs to confirm what was achieved in the present study. 

## Figures and Tables

**Table 1:** Task-oriented ADL training program.

Task	Task-Oriented ADL Training Program
Independent toileting	Moving to the front of the toilet Sitting on the toilet Pulling down the lower garment Discharging (through imitation) Performing after-care Pulling up the lower garment and tidying up Moving to the washstand Washing hands and wiping water
Face-washing, applying toothpaste, and brushing teeth	Applying water to the face Applying soap to the hands and face Rinsing the hands and face with water Wiping the face with a towel Fetching toothpaste and a toothbrush Applying the toothpaste to the toothbrush Brushing the teeth Rinsing the mouth with water Wiping the mouth with a towel
Taking out, changing upper, and lower garments	Moving to the front of the wardrobe Opening the door of the wardrobe and taking out the upper and lower garments Putting on the upper garment and buttoning up Putting on the lower garment Tidying up
Brewing and drinking tea	Taking out the coffee pot Placing water in the coffee pot and switching it on Boiling the water Placing tea in a cup Pouring the boiled water into the cup Sitting around the table to drink the tea
Cooking Ramyeon	Taking out the pan Placing water in the pan Switching on the stove to boil the water Opening the Ramyeon noodle and soup powder sachets Adding the noodle and soup powder into the boiled water Letting the Ramyeon cook Placing the cooked Ramyeon in a bowl
Dish-washing	Arranging the dishes to be washed Applying the detergent to the sponge Rubbing the dishes with the sponge Rinsing the dishes with water Arranging the dishes on the rack
Using a vacuum cleaner and moving pieces of furniture to clean a space	Bringing over the vacuum cleaner Pulling the cord out Plugging the cord Pushing the cleaner, while moving the furniture in and out of designated areas Unplugging the cord Tidying up the cleaner
Ironing a shirt on an ironing board	Preparing the ironing board Placing the shirt on the board Switching on the iron Ironing the shirt Switching off the iron Tidying up the board
Making a purchase at a nearby shop	Pushing the lift button Taking the lift to the 1st floor Walking to the shop Selecting the designated object at the shop Paying for the object Walking back to the hospital
Enjoying a simple leisure activity (table tennis, catch ball)	Playing a desired activity between table tennis and catch ball.

Abbreviations: ADL, activities of daily living.

**Table 2:** General characteristics of the participants (*n* = 30).

	Task-Oriented ADL Training Program (*n* = 15)	Conventional Occupational Therapy (*n* = 15)	*p*
Age (years)	54.4 ± 12.7	59.8 ± 8.3	0.277
Sex, *Females*, *n* (%)	4 (26.7)	3 (20.0)	0.886
BMI (kg/m^2^)	22.9 ± 1.8	23.4 ± 2.6	0.658
Affected side, left, *n* (%)	11 (73.3)	10 (66.7)	0.702
K-MMSE	26.6 ± 2.2	26.9 ± 2.3	0.740

Abbreviations: BMI, body mass index; K-MMSE, Korean version of the Mini-Mental State Examination; ADL, activities of daily living.

**Table 3:** Primary outcome variables before and after the interventions between the groups (*n* = 30).

			Task-Oriented ADL Training (*n* = 15)	Conventional OT (*n* = 15)	Group × Time Interaction F (*p*)	Main Effect of Time F (*p*)	Main Effect of Group F (*p*)
MFT	Affected	Pre	18.53 ± 3.70	18.93 ± 3.73	4.200 (0.050)	85.050 (<0.001)	0.097 (0.757)
		Post	22.93 ± 4.10 *	21.73 ± 3.08 *			
	Non- affected	Pre	26.40 ± 3.66	26.00 ± 3.44	0.085 (0.773)	22.712 (<0.001)	0.145 (0.697)
		Post	28.13 ± 3.42	27.53 ± 3.89			
BBT	Affected	Pre	29.13 ± 7.85	29.93 ± 8.23	4.411 (0.045)	11.099 (0.002)	0.174 (0.680)
		Post	34.13 ± 9.06 *	31.07 ± 5.98			
	Non- affected	Pre	39.63 ± 12.67	40.20 ± 10.31	4.388 (0.045)	7.283 (0.012)	0.110 (0.743)
		Post	43.86 ± 9.78 *	40.73 ± 10.55			

Abbreviations: MFT, manual function test; BBT, Box and block test; ADL, activities of daily living; OT, occupational therapy. * significant changes from the pre-test.

**Table 4:** Secondary outcome variables before and after the interventions between the groups (*n* = 30).

		Task-Oriented ADL Training (*n* = 15)	Conventional OT (*n* = 15)	Group × Time Interaction F (*p*)	Main Effect of Time F (*p*)	Main Effect of Group F (*p*)
K-MBI	Pre	69.27 ± 16.66	68.47 ± 13.55	1.765 (0.195)	27.272 (<0.001)	0.312 (0.581)
	Post	78.80 ± 13.15	74.13 ± 12.15			
K-SS-QoL	Pre	142.91 ± 32.81	135.78 ± 34.32	1.557 (0.222)	10.100 (0.004)	0.685 (0.415)
	Post	154.80 ± 37.69	140.93 ± 37.47			

Abbreviations: K-MBI, Korean modified Barthel index; K-SS-QoL, Korean-stroke-specific quality of life; ADL, activities of daily living.

## Data Availability

The datasets generated during the current study are available from the corresponding author upon reasonable request.
